# Chronic eosinophilic pneumonitis due to the inhalation of aerosolized face lotion: A case report

**DOI:** 10.1097/MD.0000000000025860

**Published:** 2021-05-14

**Authors:** Masafumi Shimoda, Kozo Morimoto, Yoshiaki Tanaka, Tamiko Takemura, Teruaki Oka, Kozo Yoshimori, Ota Ken

**Affiliations:** a Respiratory Disease Center, Fukujuji Hospital, Japan Anti-Tuberculosis Association (JATA), Kiyose City, Tokyo; b Department of Pathology, Kanagawa Cardiovascular and Respiratory Center, Tomioka-higashi, Kanazawa-ku, Yokohama, Kanagawa; c Department of Pathology, Fukujuji Hospital, Japan Anti-Tuberculosis Association (JATA), Kiyose City, Tokyo, Japan.

**Keywords:** chemical pneumonia, eosinophilic pneumonia, exposure, face lotion, inhalation

## Abstract

**Rationale:**

Inhalation of toxic agents can induce eosinophilic pneumonia. However, only a few case reports demonstrate that exposure to materials can induce chronic eosinophilic pneumonia (CEP). Here, we describe a rare case of CEP with mild alveolar hemorrhage due to the inhalation of aerosols from face lotion. This is the first report of eosinophilic pneumonia caused by face lotion exposure.

**Patient concerns:**

A 39-year-old woman was admitted to our hospital with cough and dyspnea for 2 months, which coincided when she started to use a new aerosolized face lotion. Laboratory findings showed high blood eosinophil levels, and chest computed tomography (CT) scans revealed bilateral peripheral consolidation and ground-glass opacity mainly in the left upper lobe. She underwent flexible bronchoscopy. Eosinophils in bronchoalveolar lavage fluid (BALF) were slightly elevated, and the gross appearance of BALF was bloody. The histological examination of the transbronchial lung biopsy showed infiltration of eosinophils and macrophages in alveolar septa with edema and without vasculitis and granuloma formation; a small number of hemosiderin-laden macrophages were also observed. An inhalation challenge test involving the face lotion was performed. Six hours after the test, the blood test showed an increased white blood cell (WBC) count, and chest radiography showed slight exacerbation. Forced vital capacity decreased the following day.

**Diagnosis:**

According to histological analysis and positive result of an inhalation challenge test, she was diagnosed with CEP with mild alveolar hemorrhage due to inhalation of aerosols from the face lotion.

**Interventions and outcomes:**

She gradually improved without medication after stopping the use of face lotion.

**Lessons:**

To the best of our knowledge, this is the first report of CEP with mild alveolar hemorrhage due to the inhalation of face lotion. Various inhaled agents, such as face lotion, can induce CEP in rare cases.

## 1 Introduction

Inhalation of toxic agents can induce eosinophilic pneumonia, which comprises a heterogeneous group of syndromes characterized by eosinophilic inflammation in the lungs.^[1–3]^ Generally, eosinophilic pneumonia is classified as acute eosinophilic pneumonia (AEP) or chronic eosinophilic pneumonia (CEP) based on the rate of disease progression, peripheral eosinophil count, imaging features, smoking history, and other factors.^[1,4]^ The most common cause of AEP is cigarette smoking, and other rare causes are as follows: exposure to dust (from fireworks, fine airborne sand, indoor renovations, or World Trade Center collapse), drug consumption (cocaine, marijuana, heroin, or crystal methamphetamine), cave exploration, smokehouse cleaning, gasoline tank cleaning, exposure to tear gas, plant repotting, or woodpile transport.^[2]^ Conversely, only a few case reports demonstrate that exposure to materials can induce CEP.^[5–7]^ Here, we describe a rare case of CEP with mild alveolar hemorrhage due to the inhalation of aerosols from face lotion. This is the first report of eosinophilic pneumonia caused by face lotion exposure.

## 2 Case presentation

**Figure 1:**
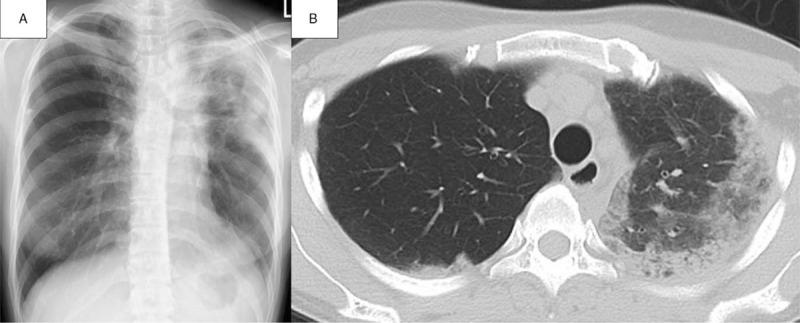
Radiography and chest high-resolution computed tomography (CT) scans. A: Pulmonary infiltrates in the left upper lobe on chest radiography. B: Bilateral peripheral consolidation and ground-glass opacity in the left upper lobe on chest CT.

A 39-year-old woman was admitted to our hospital with cough and dyspnea for 2 months. She had a medical history of bronchial asthma, allergic rhinitis, and schizophrenia for which she took aripiprazole several years previously. She had no smoking history and no remarkable allergic history. She had not used typical medicine to treat her bronchial asthma; however, a short course of salmeterol xinafoate/fluticasone was required approximately every other year. Two months prior to admission, she started to use a new face lotion (HANA ORGANIC Floral Drop), which was formulated as an aerosol spray with many organic elements (Table 1). She sprayed the lotion toward her face and inhaled the aerosols, following the recommended usage by the manufacturer. Since then, she noticed symptoms including cough and dyspnea. She went to a local doctor and was prescribed salmeterol xinafoate/fluticasone propionate and montelukast. However, her symptoms did not improve, and chest radiography showed the presence of consolidation in the left upper lung; therefore, she visited our hospital. Her vital signs and physical examination were unremarkable. The laboratory findings were as follows: white blood cell (WBC) count 10,150 cells/μL, with 46.0% polymorphic nuclear leukocytes and 36.8% eosinophils; C-reactive protein 0.04 mg/dL; serum Krebs von den Lungen-6 292 U/mL; and serum immunoglobulin E (IgE) 287 U/mL (Table 2). Chest radiography revealed pulmonary infiltrates in the left upper lobe (Fig. 1A). A chest computed tomography (CT) scan showed bilateral peripheral consolidation and ground-glass opacity mainly in the left upper lobe (Fig. 1B). The pulmonary function test showed normal findings (percent predicted forced vital capacity was 86.43% and percent predicted forced expiratory volume in 1 second was 84.1%), and inhaled short-acting β2 agonists did not improve the forced expiratory volume in 1 second. Fractional exhaled nitric oxide (FeNO) was present at a high concentration of 88 ppb.

**Table 1:** Organic elements in the face lotion.

Organic elements in the face lotion
Damask rose flower water
Propane diol
ethanol
*α*-glucan oligosaccharide
Honey
Rosemary leaf extract
Hydrolyzed coix seeds
Cucumber fruit extract
Lactic acid bacillus/radish root fermented liquid
Glycerin
Salvia sclarea
Sweet-scented geranium oil
Bitter orange flower oil
Ylang ylang flower oil
Lavender oil
Bitter orange leaf/branch oil
Orange peel oil
Citric acid, polyglycerol laurate-10
Sodium levulinate
Sodium anisate
Sucrose laurate
1,3-butylene glycol

**Table 2:** Laboratory findings of the patient.

Peripheral blood	
Hemoglobin (N: 14–18)	12.5 g/dL
Hematocrit (N: 40–48)	37.1%
RBC (N: 410 × 10^4^–530 × 10^4^)	424 × 10^4^/μL
WBC (N: 4000–8000)	10150/μL
Neutrophils (N: 48–61)	46.0%
Eosinophils (N: 1–5)	36.8%
Basophils (N: 0–1)	1.3%
Monocytes (N: 4–7)	4.5%
Lymphocytes (N: 25–45)	11.4%
Platelets (N:13 × 10^4^–35 × 10^4^)	27.3 × 10^4^/μL
Coagulation	
PT-INR (N: 0.6–1.5)	1.00
APTT (N: 24–42)	28.7 sec
Fibrinogen (N: 200–400)	371 mg/mL
FDP (N: <5.0)	<2.5 μg/mL
D-dimer (N: <1.0)	1.1 μg/mL
Serology	
C-reactive protein (N: 0–0.3)	0.04 mg/dL
IgG (N: 870–1700)	2103 mg/dL
IgE (N: 0–400)	287 IU/mL
KL-6 (N: 0–500)	292 U/mL
SP-D (N: <110)	78.4 ng/mL
Anti-CCP	Negative
Antinuclear antibody	Negative
SS-A	Negative
Anti-ARS	Negative
MPO-ANCA	Negative
PR3-ANCA	Negative
IGRA	Negative
Anti-*Trichosporon asahii* antibody	Negative
Anti-*Aspergillus* antibody	Negative
Blood biochemistry	
Na (N: 135–145)	138 mmol/L
K (N: 3.5–5)	3.9 mmol/L
Cl (N: 98–108)	104 mmol/L
BUN (N: 8–20)	8.0 mg/dL
Creatinine (N: 0.53–1.00)	0.57 mg/dL
Total protein (N: 6.7–8.3)	7.96 g/dL
Albumin (N: 3.8–5.1)	4.19 g/dL
Total bilirubin (N: 0.2–1.2)	0.6 mg/dL
AST (N: 115–359)	16 IU/L
ALT (N: 8–42)	10 IU/L
LDH (N: 119–229)	229 IU/L
CK (N: 62–287)	38 IU/L
NT-pro BNP (N: <125)	17.0 pg/mL
Tumor markers	
sIL-2 receptor (N:122–496)	554 U/mL
Bronchoalveolar lavage fluid	
Total cell count (N: 0.7 × 10^5^–2 × 10^5^)	11.36 × 10^6^/mL
Lymphocytes (N: 10–15)	13.2%
Neutrophils (N: <3)	1.8%
Eosinophils (N: <1)	8.0%
Macrophages (N: 80–90)	77.0%
CD4/8 ratio (N: 1–3)	0.3

ALT = alanine transaminase, anti-ARS = anti-aminoacyl tRNA synthetase antibodies, anti-CCP = anti-cyclic citrullinated peptide antibody, APTT = activated partial thromboplastin time, AST = aspartate aminotransferase, BUN = blood urea nitrogen, CD4/8 ratio = ratio of CD4+ to CD8+ cells, CK = creatinine kinase, Cl = chloride, FDP = fibrin/fibrinogen degradation products, Ig = immunoglobulin, IGRA = interferon gamma release assay, K = potassium, KL-6 = Krebs von den Lungen-6, LDH = lactate dehydrogenase, MPO-ANCA = myeroperoxidase-anti-neutrophil cytoplasmic antibodies, N = normal range, Na = sodium, NT-pro BNP = N-terminal probrain natriuretic peptide, PR3-ANCA = proteinase3-anti-neutrophil cytoplasmic antibodies, PT-INR = prothrombin time-international normalized ratio, RBC = red blood cells, sIL-2R = soluble interleukin-2 receptor, SP-D = surfactant protein D, SS-A = anti-Sjögren's-syndrome-related antigen A, WBC = white blood cells.

**Figure 2:**
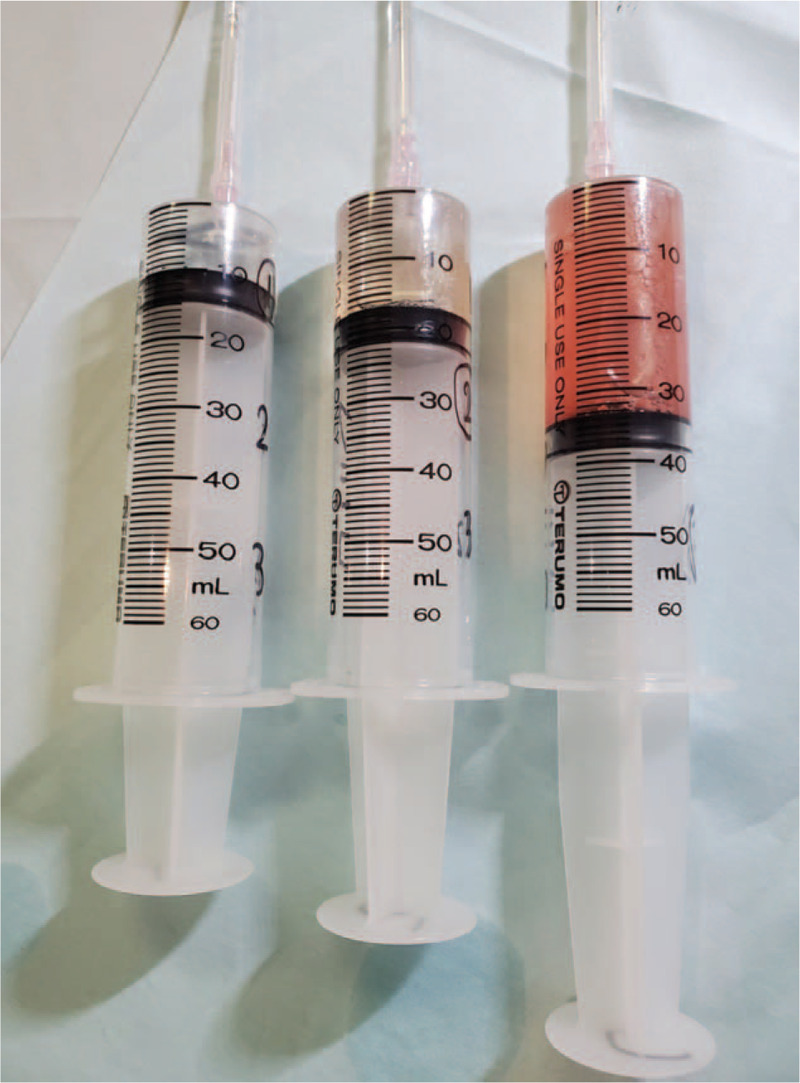
The bloody gross appearance of the bronchoalveolar lavage fluid.

**Figure 3:**
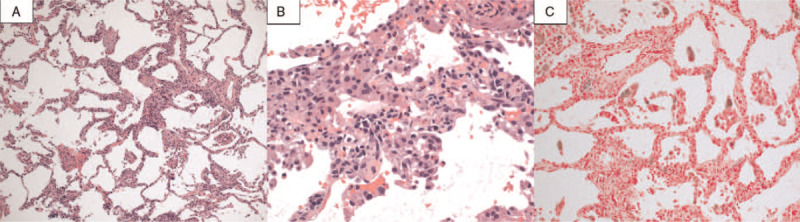
Histological examination of the transbronchial lung biopsy showed infiltration of eosinophils and multinucleated macrophages in alveolar septa with edema, slightly swollen type II pneumocytes, a small amount of organized intra-alveolar fibrinous exudate and organized foamy macrophages, without vasculitis and granuloma formation. Some hemosiderin-laden macrophages were also observed on iron staining. A: Hematoxylin–eosin (H&E) staining × 100, B: H&E staining × 400, C: Iron staining × 200.

Flexible bronchoscopy was performed because eosinophilic pneumonia due to face lotion exposure was suspected. Bronchoalveolar lavage fluid (BALF) revealed an elevated cell count (11.36 × 10^6^/mL), with 13.2% lymphocytes and 8.0% eosinophils. The gross appearance of BALF was bloody (Fig. 2). Infectious organisms were not identified on routine BALF staining or culture. The histological examination of the transbronchial lung biopsy (TBLB) showed the infiltration of eosinophils and macrophages in alveolar septa with edema and a small number of organized, foamy macrophages in intra-alveolar fibrinous exudate, without vasculitis and granuloma formation (Fig. 3A, B). Some hemosiderin-laden macrophages were also observed (Fig. 3C). We performed an inhalation challenge test with the face lotion. She sprayed the face lotion 8 times in the direction of her face and breathed deeply, which is the recommended usage. Six hours after the test, the blood test showed an increased WBC count from 8700 cells/μL with 3830 cell/μL neutrophils to 11,130 cells/μL with 6090 cell/μL neutrophils, and chest radiography showed slight exacerbation. The forced vital capacity decreased from 2.81 Liter to 2.59 Liter on the following day. Therefore, we considered that the inhalation challenge test produced a positive result. Accordingly, she was diagnosed with eosinophilic pneumonia with mild alveolar hemorrhage due to inhalation of aerosols from face lotion. Her symptoms, laboratory findings, including eosinophilia, FeNO, and radiographic findings gradually improved without medication after stopping the use of the face lotion. Peripheral eosinophils and FeNO decreased to 540 cells/μL and 39 bpp 2 months after discharge, respectively. We obtained informed consent from the patient for the publication of study.

## 3 Discussion

We present a rare case of eosinophilic pneumonia due to the inhalation of aerosols from face lotion. The patient had evidence of eosinophilia according to laboratory findings, slightly elevated eosinophils in BALF, and eosinophilic infiltration and mild alveolar hemorrhage in alveolar septa according to histopathologic findings. Systematic chronic disease progression met the diagnostic criteria for CEP^[3]^ but did not meet the diagnostic criteria for AEP.^[1]^ Generally, contact dermatitis is the outcome of skin lotion allergy,^[8]^ which is common; there is no report of eosinophilic pneumonia caused by skin lotion. The patient was diagnosed with CEP with mild alveolar hemorrhage related to skin lotion use because of a positive challenge test involving inhalation of the skin lotion and an improvement in the disease after stopping skin lotion use.

The patient's symptoms fulfilled the diagnostic criteria for drug- or toxin-induced eosinophilic pneumonia. The criteria are as follows:

1. the presence of simple, acute, or CEP according to diagnostic criteria;
2. exposure to a potential candidate drug or toxin in an appropriate time frame;
3. the exclusion of other causes of eosinophilic pneumonia, such as fungal or parasitic pneumonia;
4. clinical improvement after cessation of exposure to the drug or toxin; and
5. recurrence upon rechallenge with the drug or toxin.^[9]^

Our patient met all the criteria; however, it was uncertain which element in the face lotion was the causative agent because each element could not be examined individually, and her allergy tests, including total and various specific IgE detection, showed negative results. Considering that she had eosinophilia and a high FeNO level, which is a useful biomarker of eosinophilic inflammation,^[10]^ exposure to 1 or multiple elements in the face lotion might have triggered the development of eosinophilic pneumonia and eosinophilia.

CEP due to an inhaled agent is rare, and only 3 previous reports showed that exposure to isocyanates, acrylic airbrush paints, and *Schizophyllum* induced CEP, respectively.^[5–7]^ In our case and the other CEP reports, several atypical findings were observed. Our patient and 1 patient from a previous report had normal IgE levels,^[6]^ and 2 patients from previous reports showed atypical findings on CT, such as consolidation predominance in the lower lobes^[5]^ or a honeycomb appearance.^[7]^ Regarding treatments, a patient with CEP due to exposure to acrylic airbrush paint did not respond to systemic steroids,^[5]^ while 2 other patients improved with systemic steroids^[6]^ and inhaled steroids,^[7]^ respectively. Our patient did not require any treatment other than avoiding face lotion use. Generally, the response of patients with CEP to systemic steroids is dramatic and fairly rapid, and less than 10% of patients with CEP show spontaneous resolution without treatment.^[11]^ Considering this point, CEP due to exposure via inhalation might follow an atypical course compared to CEP due to other reasons.

Interestingly, our patient had mild alveolar hemorrhage, which was not observed in other previous cases of CEP.^[5–7]^ Generally, patients with eosinophilic pneumonia do not develop alveolar hemorrhage.^[1]^ Patients with lung injury due to inhaled toxicants, such as waterproofing spray and toxic chemicals, sometimes have evidence of alveolar hemorrhage in BALF.^[12,13]^ The mechanism underlying the inhalation of fluorescein in waterproofing spray induces macrophage infiltration, thickening of the alveolar septum, and pulmonary collapse, which are related to alveolar hemorrhage.^[14,15]^ Other inhaled materials, such as mycotoxins, cadmium, and marijuana, are also reported to cause alveolar hemorrhage.^[13,16,17]^ The face lotion involving in our case included many organic elements, such as oils, solvents, multivalent alcohols, and preservative agents, which were derived from plants. Although those elements have not been associated with any pulmonary adverse events, the safety of inhaling these substances is unclear, and 1 or some elements might induce a toxic reaction. Indeed, inhalation of marijuana can induce alveolar hemorrhage, even though it is derived from plants.^[16]^ Hence, the mechanisms of eosinophilic pneumonia due to the inhalation of face lotion in our case might be associated with not only an eosinophilic reaction but also a mild toxic chemical reaction because the patient's BALF and histopathologic findings indicated mild alveolar hemorrhage.

This report has several limitations. We could not investigate an allergic response to each element in the face lotion; therefore, which elements were the causative agents could not be revealed. We also could not determine the time at which eosinophilia developed, whether it was related to eosinophilic pneumonia, or its cause.

## Author contributions

**Conceptualization:** Masafumi Shimoda.

**Data curation:** Masafumi Shimoda, Kozo Morimoto, Yoshiaki Tanaka, Tamiko Takemura, Teruaki Oka, Kozo Yoshimori.

**Formal analysis:** Masafumi Shimoda.

**Investigation:** Masafumi Shimoda.

**Methodology:** Masafumi Shimoda, Kozo Morimoto, Yoshiaki Tanaka.

**Project administration:** Ota Ken.

**Supervision:** Kozo Yoshimori, Ota Ken.

**Visualization:** Masafumi Shimoda.

**Writing – original draft:** Masafumi Shimoda.

**Writing – review & editing:** Masafumi Shimoda, Kozo Morimoto.
